# Factors Associated with Outpatient Satisfaction in Tertiary Hospitals in China: A Systematic Review

**DOI:** 10.3390/ijerph17197070

**Published:** 2020-09-27

**Authors:** Yuping Li, Weijuan Gong, Xiang Kong, Olaf Mueller, Guangyu Lu

**Affiliations:** 1 Department of Neurosurgery, The Clinical Medical College of Yangzhou University, Yangzhou University, Yangzhou 225009, China; yupingli@yzu.edu.cn; 2 Nursing School of Yangzhou University, Yangzhou University, Yangzhou 225009, China; wjgong@yzu.edu.cn; 3 Department of Gynecology and Obstetrics, The Clinical Medical College of Yangzhou University, Yangzhou University, Yangzhou 225009, China; xkong@yzu.edu.cn; 4 Heidelberg Institute of Global Health, Medical School, Ruprecht-Karls-University, INF 130.3, 69120 Heidelberg, Germany; Olaf.Mueller@urz.uni-heidelberg.de; 5 Institute of Translational Medicine, School of Medicine, Yangzhou University, Yangzhou 225001, China; 6 Jiangsu Key Laboratory of Integrated Traditional Chinese and Western Medicine for Prevention and Treatment of Senile Diseases, Yangzhou University, Yangzhou 225001, China; 7 Department of Preventative Medicine, Medical College of Yangzhou University, Yangzhou University, Yangzhou 225009, China

**Keywords:** patient satisfaction, doctor-patient communication/interaction, quality of care, health services research, evidence-based medicine

## Abstract

Outpatient care is made up of medical procedures, tests, and services that can be provided to the patient in a setting that doesn’t involve an overnight hospital stay. In China, tertiary hospitals are medical services centers of health care systems, and some tertiary hospitals had more than 20,000 outpatient visits per day. However, a systematic review of existed evidence on factors influencing the outpatient satisfaction in tertiary hospitals in China could inform the efforts and does not yet exist. Therefore, in order to better understand the outpatient satisfaction provided by tertiary hospitals in China, we carried out a systematic review following PRISMA guidelines. Studies reporting on the level of and factors associated with outpatient satisfaction in Chinese tertiary hospitals were systematically searched in both Chinese and English electronic databases. A total of 36 articles reported 35 studies that met the inclusion criteria. Out of these eight were household surveys covering 12,119 residents, and another 27 directly interviewed 45,930 outpatients during their hospital visits from 185 hospitals. The included studies generally used self-designed questionnaire and indicated there is a lack of standardized questionnaire for investigating outpatient satisfaction in China. The outpatients showed the highest satisfaction with the doctors and nurses and the lowest satisfaction with the hospital hygiene and outpatient procedures, especially with the long waiting time. The socio-demographic characteristics (e.g., age, marital status, income and education levels), professional skills and service attitudes of medical staff were reported to be associated with outpatient satisfaction. The results indicated that in China, the outpatient satisfaction can be largely improved. Firstly, the attitude of medical service providers, especially the pre-diagnosis nurses, registration officers, and pharmaceutical counters should be improved. Furthermore, to shorten the waiting time, policies should be developed to guide patients with common diseases and slight discomforts to community health systems to alleviate the overload in tertiary hospitals. Considering the strained relations between the doctors and patients in the clinical practice, improving patient satisfaction in China deserves more attention and research.

## 1. Introduction

**Figure 1:**
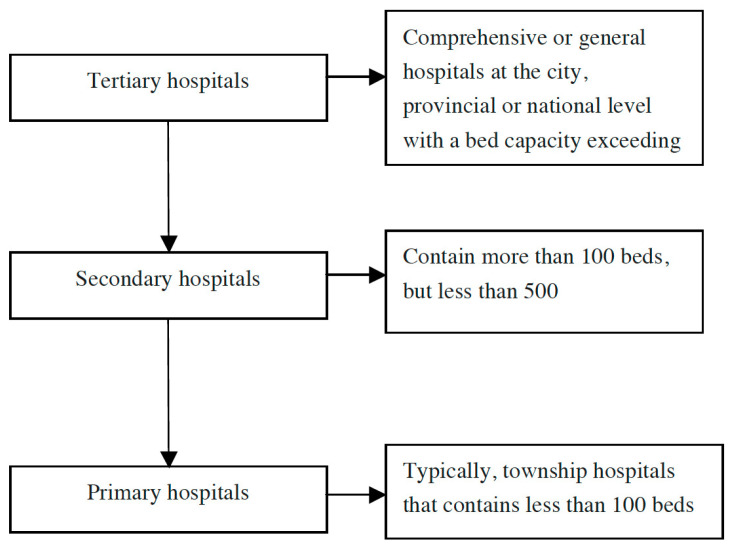
Classification of Chinese hospitals (Hospitals in China are organized according to a three-tier system including primary, secondary, or tertiary levels.).

Hospitals in China are organized according to a three-tier system (designated as primary, secondary or tertiary institutions) that recognizes a hospital’s ability to provide medical care and medical education and to conduct medical research (Figure 1). Tertiary hospitals are medical services centers within the region and play a key role in the medical and health service system [1]. Patients in China prefer tertiary hospitals when seeking medical services because tertiary hospitals represent the best medical resources, including both outpatient and inpatient care. According to the statistics from the National Health and Family Planning Commission of the People’s Republic of China, in 2014, 2.6 billion patients visited Chinese hospitals, 46% of which visited tertiary hospitals [2].

Outpatient care is made up of medical procedures, tests, and services that can be provided to the patient in a setting that doesn’t involve an overnight hospital stay. Some tertiary hospitals in China had more than 20,000 outpatient visits per day, and doctors must see more than 100 patients over the course of 1 day [3]. In such a context, violence against healthcare staff during outpatient visits frequently occurs [4,5]. According to the data from the Chinese Hospital Association, at a rate of once every two weeks per hospital, patients or their relatives have attacked medical staff in China [6]. Moreover, tertiary hospitals were found to have more medical conflicts and lower patient satisfaction compared with primary hospitals [7].

In China, patient satisfaction surveys have gained increasing attention, and many hospitals have sought to improve the quality and the experience of outpatient care over the past decade. However, the majority of the previous surveys on outpatient satisfaction were conducted in individual hospitals and, therefore, were aimed at the development of that particular hospital [8,9,10,11]. Some researchers have analyzed outpatients with diverse types of diseases from different hospitals in one region [7,12,13]; however, the results were still limited to the development of the specific area. Previous studies suggested the conflicts between Chinese doctors and patients at outpatient services has been largely attributed to the extremely poor quality of the doctor-patient relationship [4,5,14,15]. But, a systematic review of existed evidence on factors influencing the outpatient satisfaction in tertiary hospitals in China could inform the efforts and does not yet exist. Therefore, the objective of this study is to develop a comprehensive understanding of outpatient satisfaction in tertiary hospitals and to conduct a critical review of the measurement tools used to evaluate the outpatient satisfaction in China. This review is expected to provide meaningful and essential sources of information to identify gaps, harmonize the patient–doctor relationship, and develop effective action plans for quality improvement in healthcare organizations in China.

## 2. Materials and Methods 

This systematic review followed the preferred reporting items for systematic reviews and meta-analysis (PRISMA) checklist [16]. 

### 2.1. Study Inclusion and Exclusion Criteria

Studies that have evaluated the levels or associated factors regarding outpatient satisfaction in Chinese tertiary hospitals are included, with no language or study design restrictions. Participants of the studies could be the outpatients or residents who had experience receiving outpatient services in a tertiary hospital. Outpatients refers to patients receiving medical care without an overnight stay in the hospital.

Studies conducted in non-tertiary hospitals, community health centers. or village clinics were excluded. Studies were excluded if the participants were not Chinese. We also excluded studies involving participants with one specific disease from one specialized outpatient department. Reviews, comments, editorials, or letters were also excluded.

### 2.2. Search Strategy and Selection Criteria

The primary data sources for this review include English electronic databases, including MEDLINE, Cochrane, PubMed, Embase, and Web of Science, and Chinese electronic databases, including the Chinese National Knowledge Infrastructure and VIP Database. No language restrictions were applied, and the databases were searched from their inception until 28 February 2018. The search used combinations of the terms patient, satisfaction and China as both MeSH headings and key or free text words and included a wide range of derivations to ensure as wide a search strategy as possible. A list of the detailed search strategy used is available online as supplemental material (Supplementary Material 1). A grey literature search was explored by search conference abstracts or papers, hard to find studies, reports, or dissertations in university library catalogs. In addition, the reference lists of the included articles were manually screened for potentially relevant studies that could have been missed during the electronic database search. 

### 2.3. Data Extraction and Analysis

The electronic reports identified were imported into the reference manager Endnote and duplicates removed. Each paper was assessed in two phases: first by screening title and abstract, and then by full-text review to ensure they met the inclusion criteria. The first assessment was done by two reviewers (Y.L. and G.L.). The second assessment was done by G.L. and Q.C. An additional reviewer (O.M.) settled any discordance between the reviewers.

It was decided a priori that if the data from different studies were sufficiently homogeneous and the combination of the collected data was justifiable, a meta-analysis would be conducted. However, if the results are too heterogeneous, we will describe all study outcomes using a narrative analysis based on primary objectives of studies to clarify study results and draw conclusions [16]. The degree of heterogeneity was evaluated on the characteristics of participants of the study and tools used in outpatient satisfaction evaluation.

We developed a standardized data collection form based on the Cochrane Consumers and Communication Review [17]. The data from the included articles were extracted independently by two reviewers (G.L. and Y.L.). For each article, we extracted the data for the hospital backgrounds, sample size, participant information, sampling methods, assessment instrument, and response rate. We also extracted the key findings from the included articles [17]. Disagreements were solved by consensus.

If the studies only evaluated the levels of outpatient satisfaction, we reported the satisfaction levels. If the studies also analyzed, the associated factors of outpatient satisfaction, we extracted the main findings on the relationship between outpatient satisfaction and its influencing factors. The influencing factors were categorized as follows: patient factors, medical staff factors, the medical environment, and process management. 

### 2.4. Quality Assessment

Considering the fact that the majority of the satisfaction surveys are cross-sectional studies, the Newcastle-Ottawa scale, modified for cross-sectional studies, was used as a tool for risk of bias assessment of all selected articles (Supplementary Material 2). This scale addresses 3 domains (selection, outcome, and comparability), and the studies could be awarded 1 star for each factor in the first 2 categories (sum of 5 stars) and 2 stars for each factor in the comparability section. The sum of the stars, up to a maximum of 7, reflected the overall quality rating of each study. The higher the number of stars, the higher the quality rating. Two reviewers (G.L., Y.L.) separately completed the quality assessments of the selected studies. Once again, disagreements were solved by consensus.

## 3. Results

**Figure 2:**
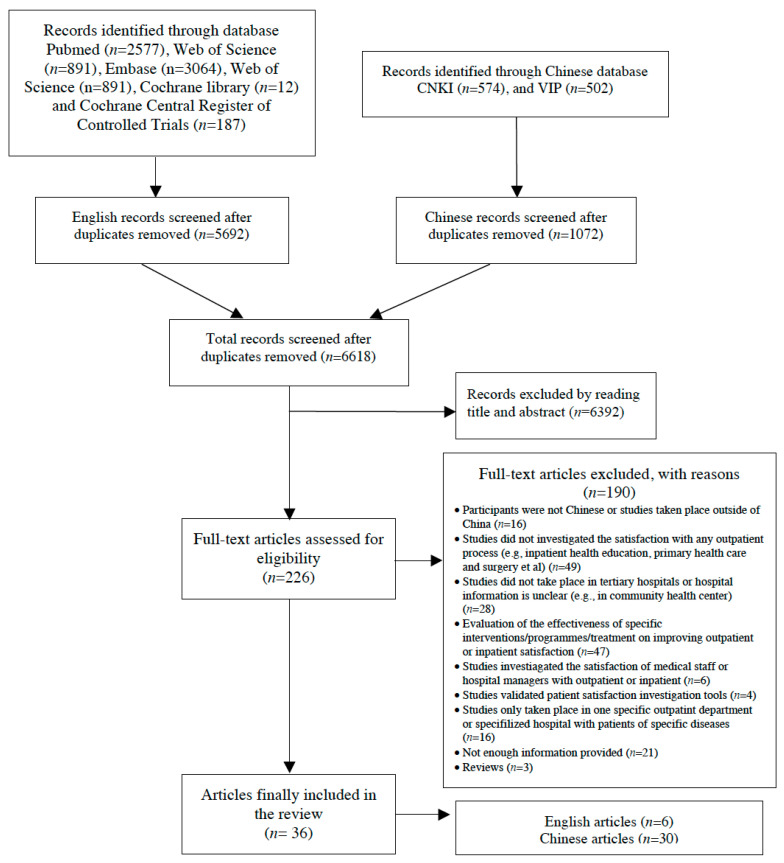
Selection Process Flow Diagram [52,53].

We identified 6507 citations by the literature search. The broad selection of articles by title and abstract led to the retrieval of 226 potentially eligible studies. After a full-text review of these 226 studies, 190 studies were excluded with the reasons (Supplementary Material 3). A total of 36 articles reported 35 studies that met the inclusion criteria of this review [7,15,18,19,20,21,22,23,24,25,26,27,28,29,30,31,32,33,34,35,36,37,38,39,40,41,42,43,44,45,46,47,48,49,50,51]. All studies were published in peer-reviewed journals. Six of the studies were published in English [7,15,18,26,28,31], and 30 of the studies were published in Chinese. The selection process is summarized in Prisma searching flow diagram (Figure 2). 

### 3.1. Characteristics of Included Studies

This study included patients with a variety of diseases from the outpatient departments in tertiary hospitals in different areas of China. The degree of heterogeneity was evaluated based on the basic information of include studies. The included studies have great differences in age, living place (urban or rural) of patients, and the tools used in outpatient satisfaction evaluation, which indicated that the degree of heterogeneity too great for any quantitative analysis. The characteristics of included studies were reported in Table 1, including study context, number of participants, participant information, sampling methods, and study design. 

All of the studies were published after 2000, and 30 out of 35 studies (36 papers) were published after the year 2010. All the included studies used survey designs, eight of which were household surveys, covering resident populations up to 12,119. Another 27 studies directly interviewed the outpatients during their visits to the hospital. These 27 hospital-based studies reported data from 185 hospitals with 45,930 outpatients in nearly the whole of China. Eighteen out of these 35 studies analyzed the relationship between outpatient satisfaction and the influencing factors [7,12,18,19,20,21,22,23,24,25,26,27,28,29,30,31,32,33,34], while another 17 only descriptively reported the most satisfactory or dissatisfactory factors during outpatient service. 

### 3.2. Measures of Outpatient Satisfaction and the Study Quality

In terms of the methodology, the studies primarily used simple survey instruments to investigate the outpatient satisfaction. The instruments were predominantly self-designed questionnaires, either adjusted based on an existing questionnaire, such as the Inpatient Satisfaction Questionnaire (IPSQ), or were designed by the researchers. The Likert-type scale (3-point, 5-point or 7-point scales) was primarily employed to measure the attitude of the outpatients (33/35, 94.3%). 

Table 2 listed the quality appraisal for the included studies. Of these 35 studies reported by 36 papers, in terms of quality, 8 were very good, 20 were of satisfactory, and 7 were unsatisfactory. The studies published in English were generally credited with better study quality. Of the 27 hospital-based on-site studies, 21 studies used random sampling. Although the sample size of the included studies is relatively large, only about one-third of the studies reported the justification for the sample size. The majority of the studies (*n* = 32) reported the response rate, of which over 95% (30/32, 96.8%) studies reported the response rate larger than 85% (Table 3). It indicated satisfactory response rates of included studies.

### 3.3. Overall Satisfaction Level

The assessment instrument, response rate and overall findings on levels of outpatient satisfaction were reported in Table 3. The majority of the studies reported a high level of outpatient satisfaction, ranged from 76.9–94.6%. Although the factors each study investigated varied, 22 out of 35 studies (62.9%) provided information on which factors were the most satisfactory and unsatisfactory (Table 3 and Table 4). The most frequently investigated factors include the satisfaction with the following five aspects: the professional skills and service attitude of the medical staff (doctors and nurses, 19/22, 86.4%), the service attitude of the staff in other departments or administrative offices (e.g., the payment office or pre-diagnostic counters, 18/22, 81.8%), the hospital hygiene and facilities (18/22, 81.8%), the waiting time (15/22, 68.2%), and medical costs (13/22, 59%). Waiting time is the most frequently investigated factor in the aspect of outpatient process and management, followed by diagnosis and treatment process (12/22, 54.5%), easy access to hospital and registration (5/22, 22.7%), medical needs being met (4/22, 18.2%), and ease of complain (3/22, 13.6%).

The outpatients showed the highest satisfaction with the doctors and nurses (yellow color) and showed the lowest satisfaction with the hospital hygiene and outpatient procedures (blue color) (Table 4). A total of 78.9% (15/19) studies reported that the professional skills and service attitude of the medical staff (including doctors and nurses) were the most satisfying factors during outpatient services. Moreover, only 2 of the 10% (2/19) studies reported dissatisfaction with the people at the hospital, and it is important to notice their dissatisfactions were with the pre-diagnosis counters and the registration/payment officers instead of the doctors and nurses. Moreover, 60% (9/15) studies found that waiting time was the most dissatisfied factor. 

### 3.4. Relationships between Outpatient Satisfaction and Influencing Factors

Eighteen of the 35 studies (51.4%) analyzed the relationships between outpatient satisfaction and its influencing factors (Table 5).

#### 3.4.1. Patient Social-Demographic Factors

Twelve of the 18 studies (66.7%) investigated the association of socio-demographic factors with outpatient satisfaction. 83.3% of studies (10/12) found that the main socio-demographic characteristics (including sex, age, occupation, monthly income, residence, and marital status) of outpatients were related to satisfaction to varying degrees. While, 16.7% of studies (2/12) reported there were no significant differences in the sex, age, marital status, and occupation (*p* > 0.05) of the outpatients regarding outpatient satisfaction [25,33].

The satisfaction of the Chinese elderly outpatients were found to be significantly higher than those of the young and middle-aged outpatients. However, the elderly outpatients were less satisfied in the domain of the hospital information experience than young and middle-aged outpatients [19]. The findings on genders were controversial. Two studies have reported that, females are more likely to be satisfied regarding the waiting time [7,31]. In contrast, another study reported that male outpatients were more likely to be highly satisfied with doctors than female outpatients [12]. Four studies reported that patients who were married seemed to be more satisfied, and the divorced or widowed patients had lower odds of high satisfaction [7,12,22,29]. Moreover, higher incomes and higher education levels were both associated with lower odds of satisfaction [7,22,23,25,31]. Two studies reported that insured patients were much more likely to be satisfied [7,26]. One study reported that health-related knowledge was positively associated with outpatient satisfaction [27]. 

#### 3.4.2. Medical Staff Factors

Seven of 18 studies (38.9%) investigated the association of staff factors with outpatient satisfaction. Studies have consistently reported that the professional skills and service attitudes of doctors and nurses were significantly associated with the overall outpatient satisfaction. Here, professional skills refer to the diagnosis and treatment skills. One study found that the time spent in communication with doctors, and degree of careful inquiry by the doctors, and the degree of the clarification by the doctors in explaining the diseases were associated with outpatient satisfaction [20]. Moreover, a trusting doctor–patient relationship and the length of communication time with doctors were found to be major factors associated with the overall satisfaction of the elderly outpatients [19,26]. It is important to notice that outpatients showed significantly lower satisfaction with administrative officers (e.g., payment officers) compared to medical providers (doctors and nurses) [30]. 

#### 3.4.3. Hospital Indoor Hygiene, Facilities, and Process Management Factors

Ten of 18 studies (55.9%) investigated the association of the hospital indoor hygiene, facilities, and process management with outpatient satisfaction. The specific aspects in this domain investigated include hospital indoor hygiene, waiting time, reputation of the hospital, management of the outpatient procedure, and the medical costs. Seven of 10 (70%) studies reported that these factors were associated with outpatient satisfaction. Waiting time was found to be associated with outpatient satisfaction in four studies [7,21,32,33]. While one population-based survey reported that the waiting time is not associated with outpatient satisfaction [27]. For elderly patients, the existence of channels for praise and complaints (OR = 1.39) was a major factor associated with the overall satisfaction. Moreover, payment methods were associated with the outpatients’ satisfaction [19,26]. 

## 4. Discussion

This is the first systematic review that has investigated the levels of outpatient satisfaction and its influencing factors in Chinese tertiary hospitals. The most frequently investigated domains included patient demographic characteristics, medical and administrative staff professional skills and attitude, hospital hygiene, and outpatient process aspects. Our paper demonstrated that outpatient satisfaction in Chinese tertiary hospitals is associated with patient social-demographic factors (age, gender, and marital status, income levels and educational levels), professional skills and service attitudes of medical staff, and waiting time. The Chinese outpatients generally showed the highest satisfaction with the professional skills and attitudes of the doctors and nurses, but the lowest satisfaction with the hospital management and environmental aspects. The findings of this study provide important insights into understanding the levels of outpatient satisfaction and the associated factors in tertiary hospitals in China. These findings are essential to improve outpatient satisfaction, increase the quality of care, strengthen the doctor–patient relationship, and further implement health reform in China.

The majority of the studies were published in this decade. This also reflects the fact that, while international research on patient satisfaction in healthcare has grown tremendously in the past three decades, research concerning outpatient satisfaction and its influencing factors in China started late and has been increasing in the recent decade. 

### 4.1. Methodological Issues and Study Quality

Although the majority of the studies in this review were of a satisfactory in quality, the review highlighted some general quality issues of the studies on outpatient satisfaction surveys. First, there is no standardized questionnaire for investigating outpatient satisfaction in China. All studies used self-designed questionnaires based on the research purposes or adjusted existing tools (e.g., the IPSQ). One large study developed and validated an outpatient satisfaction questionnaire for the Chinese population in 2012 [54]. However, it seems to be of limited use in use, as it may not apply to different levels of hospitals. Although it is well acknowledged that careful, reliable, and valid measurement of outpatient satisfaction is required, internationally acknowledged tools for achieving this objective have not been fully developed [55]. Some established tools of patient satisfaction are still in limited use because of the lack of suitability when the context changes [56]. Second, there is little standardization of the procedures for data collection and analysis. Some studies have analyzed the correlation between outpatient satisfaction and its influencing factors, while others have just reported the satisfaction scores on the investigated factors. Besides these two aspects, the included studies in this review reported representative samples from over 30 provinces in China (34 provinces total), which increased the external validity of the findings.

### 4.2. Overall Satisfaction 

Tertiary hospitals in China usually obtain financial investment in buildings, equipment, and medical resources from central and local governments and thus are expected to provide high-quality services. The results of our study demonstrated that outpatients generally show a high level of satisfaction with outpatient services in tertiary hospitals. Moreover, our study found that the outpatients showed the highest satisfaction with the professional skills and service attitude of the doctors and nurses. This finding did not support the traditional explanation regarding outpatient dissatisfaction, which was largely attributed to doctors who do not provide sufficient time to explain to or communicate well with the patients. 

To improve outpatient satisfaction, Chinese hospitals have spent time and effort improving the service attitudes of doctors and to improve the doctor–patient relationship. This was reflected by the finding in our study that outpatients are generally satisfied with the doctors regarding professional skills and service attitudes. In fact, how to maintain the good services of doctors in tertiary hospitals will be the next challenge, as doctors in tertiary hospitals (top-level hospitals) are generally overloaded. A 2014 national survey showed that 92% of the doctors in tertiary hospitals work overtime, and 72% of the doctors who have worked more than 60 h a week on average in tertiary hospitals in China [57]. 

In some studies, patients complain about the service attitude of the administrative staff working at the payment counters, registrations, and pre-diagnostic guiders. To deal with this overcrowding of the hospitals, tertiary hospitals usually set up “pre-diagnosis counters”. Pre-diagnosis counters perform triage, conduct pre-diagnoses to help patients register appropriately, and facilitate clinic processing, thereby effectively shortening the wait time and improving patient satisfaction 14. Therefore, patients usually have first contact with these staff members. However, compared with doctors, these staff members who deal with patients firstly rarely receive patient-related training on communication skills. Similar findings were noted in previous studies, wherein the service provided by pre-diagnosis counters had a strong effect on patient satisfaction [58]. Therefore, it is important to improve the service quality and attitude of the subsidiary departments. Outpatients also showed most dissatisfaction with medical costs, but this could also be attributed to the fact that outpatients in tertiary hospitals usually suffered more severe illnesses [59].

### 4.3. Patient Social-Demographic Factors

Our review indicated that the socio-demographic characteristics of outpatients were related to satisfaction to varying degrees. It is interesting to notice that a higher income was associated with lower outpatient satisfaction in China, and a similar pattern was also seen for those with a higher education level, which was associated with lower outpatient satisfaction [12,23,25,31]. This can be explained by the fact that the patients with higher levels of education and incomes usually have higher social status; therefore, their expectations are higher. 

Many prior studies demonstrated that gender, income, and education have all shown inconsistent effects on satisfaction [60,61,62,63,64]. The evidence on the influence of gender on patient satisfaction levels remains controversial in this study. Some studies have reported that women are more satisfied than men with the medical care received, and some report that women are more critical of medical care than men. In a meta-analysis of 110 studies of patient satisfaction, no average difference in satisfaction with medical care between women and men was found [61].

Our study found that outpatient satisfaction was associated with age and Chinese elderly outpatients were found to be more satisfied than young and middle-aged outpatients. This pattern is consistent with findings of studies in many other courtiers [60,61,62,63]. However, the elderly outpatients were less satisfied in the domain of the hospital information experience, such as online registration and modern payment methods (e.g., Alipay). Therefore, with the fast development of hospital information technology, related technical assistance should be provided to elderly patients to facilitate their adaptation to new developments. 

Consistent with previous findings, the results of this study showed that patients who were married seemed to be more satisfied, but the divorced or widowed patients had lower odds of high satisfaction. Therefore, based on these findings, several studies have indicated that young and middle-aged adults who are married and have a lower level of education and less income seem to have the highest level of satisfaction compared to their counterparts [7,12]. 

### 4.4. Medical Staff Factors

Our review demonstrated that the patient–doctor relationship is significantly associated with the overall patient satisfaction. Especially for the elderly outpatients, a trusting doctor–patient relationship and the length of communication time with doctors were suggested as major factors associated with the overall satisfaction. In fact, doctor–patient communication in China has gained increasing attention as the relationship between patients and health care providers has sharply deteriorated over the past decade [65]. The doctor–patient communication skills training programs were firstly introduced in China in 2003, and according to the recent study, less than 50% of Chinese medical schools include the doctor–patient communication skills training programs in the clinical medical curriculum [66]. Although there are multiple reasons behind the deterioration of the doctor-patient relationship in China in the past decade, practical measures to improve doctors’ caring competence and interaction skills with patients by providing qualified doctor-patient communication skills training programs both in the medical schools and in hospitals are crucial to improve the doctor-patient relationship [67]. 

### 4.5. Process Management Factors 

The results of this study show that patients complained frequently about the long wait time, and the wait time was negatively correlated with outpatient satisfaction scores. These findings are in line with those of previous studies done in many countries [68,69]. More patients in China are increasingly likely to visit tertiary hospitals even in cases that are not serious or complex because tertiary hospitals usually have better medical resources. This is reflected by the utilization rate of tertiary hospitals, which has risen to 100.5% in China in 2008 [70]. A previous study has suggested that outpatients were reasonably satisfied if they waited no more than 37 min when arriving on time [71]. However, considering that the study was conducted two decades ago, the time threshold may do not apply to the current metropolis rhythm. Thus, further studies are needed to provide a better understanding of the current acceptable wait time. 

The methods to reduce the wait time are challenging to implement in the current health system (e.g., increasing resources, such as staffing). However, improving the waiting experience may act as an effective way to improve the satisfaction. For example, providing a clean, comfortable environment and providing more transparent information about available healthcare services and department-related health education during the wait time. It is also important to point out that providing enough skilled doctors or general practitioners to strengthen the primary care institutions would fundamentally improve the long wait time and overcrowded situation. As a direct consequence of the increasing number of outpatients, doctors must limit their communication time to meet huge needs. Thus, it is not difficult to imagine that patients complain about the short communication time with doctors. 

Our review suggested that the outpatient process was associated with outpatient satisfaction. Process matters in healthcare and a process improvement can be successful in reducing the wait time and increasing patient satisfaction [71,72,73,74]. The Chinese government has tried to implement process reforms to increase patient satisfaction [75]. A full range of healthcare improvement requirements were established in 2015. These were designed to give patients a tangible sense of improvement in the health system, including promoting a service appointment system, optimizing the ward structure, and enhancing the health information system, and harmonizing the patient–doctor relationship to regain patient trust. 

### 4.6. Limitations of the Study

Although this study provided a comprehensive review of studies on the satisfaction of outpatients in tertiary hospitals from over 30 provinces in China. This study has several limitations. First, the included studies are cross-sectional studies, which limited the ability to draw causal conclusions. Moreover, the majority of the surveys were conducted in hospitals through face to face interviews, thus may bring the bias in answering questions as the patients may indicate higher satisfaction during their health-seeking process. While, the population-based survey also faces the recall bias from residents who had experience receiving outpatient services in a tertiary hospital. Furthermore, the heterogeneity of methodology and study settings difficult the pooling of data. Additionally, there is a lack of standardized outpatient satisfaction questionnaire, core aspects related to the outpatient satisfaction have been studied differed in included studies. Lastly, subgroup analysis of satisfaction of elderly patients was planned at the beginning, while could only be done in future study because of limited information.

## 5. Conclusions

Considering the contributions of various subtypes of satisfaction to the overall satisfaction, the results indicated that in China, the outpatient satisfaction can be largely improved. Our results suggest that, to increase satisfaction, the service attitudes of other medical service providers, including pre-diagnosis nurses, registration officers, and pharmaceutical counters, should be greatly improved. Moreover, to shorten the waiting time, policies are needed to guide patients with common diseases and slight discomforts to community health systems to alleviate the overload in tertiary hospitals. The establishment of a standardized questionnaire to investigate the outpatient satisfaction is needed. In summary, routine monitoring of patient satisfaction is needed and will help policy makers improve the quality of the Chinese healthcare system. Especially considering the strained relations between the doctors and patients in the current clinical practice, improving patient satisfaction in China deserves more attention and research.

## Figures and Tables

**Table 1:** Characteristics of included studies (*n* = 35).

Author and Year	Study Context	Number of Participants	Participant Information	Sampling Methods	Study Design
Wenya, Yu et al., 2016	11 tertiary hospitals in Shanghai	1050 outpatients from different clinical departments with varying diseases	Male: 439 Female: 532 Age was normally distributed, with the most prevalent age group 30–39 (24.41%).	Random selection	cross-sectional survey
Liyang, Tang. 2011	A household survey in 17 provinces	3424 residents	Urban residents: 3209 (93.76%) Rural residents: 34 (6.24%)	NR *	cross-sectional survey
Jing Sun et al., 2017 Laiyang, Wu et al., 2016 †	136 tertiary hospitals from 31 provinces	27,475 outpatients, of which 3923 are senior outpatients older than 60	Male: 8792 Female: 18,683	Random selection and convenient sampling	cross-sectional survey
Jay Pan et al., 2015	Survey with residents who had visited hospitals in the past 2 weeks	6393 residents	2621 male, 3772 female Mean age: 57.3 y	A multistage cluster sampling	cross-sectional survey
Jinghua, Li et al., 2016	Household survey in health care facilities in Jiling Province	68 residents had at least one outpatient visit in the past 2 weeks in tertiary hospitals.	29 male 39 female Age range: 45–64 y;	A multi-stage stratified sampling	cross-sectional survey
Jinzhu, Xie et al., 2017	Three tertiary hospitals in Hubei Province	300 outpatients	NR	NR	cross-sectional survey
Chunlei, Han et al., 2012	One Tertiary hospital in Shandong Province	338 outpatients	Male 148 Female 190	A stratified random sampling	cross-sectional survey
Qing Lu et al., 2016	3 public hospitals in Beijing	318 outpatients	Male: 137 Female: 181	Random sampling	cross-sectional survey
Jing Zhao et al., 2016	A tertiary hospital	197 outpatients	Male: 108 Female: 89 Mean age: 43.3 ± 7.3	Random sampling	cross-sectional survey
Wenlong, Hu et al., 2007	2 tertiary hospital in Xuzhou	386 outpatients	Male: 253 Female: 132	Random sampling	cross-sectional survey
Yanxia, Yang et al., 2015	A tertiary hospital	1998 outpatients	1998 outpatients Male: 993 Female: 995 Aged range: 20–70	NR	cross-sectional survey
Rong Xu and Xinzhen, Jing. 2004	A tertiary hospital in Guangdong Province	304 outpatients	304 outpatients Male: 120 Female: 175	NR	cross-sectional survey
Junjie, Sun and Shuangqing, Li. 2018	A tertiary hospital in Sichuan Province	185 outpatients	Male: 68 Female: 117	Random sampling	cross-sectional survey
Zhanwei, Zhou et al., 2011	A tertiary hospital	363 outpatients	Male: 211 Female: 152 Mean age: 45.3 ± 11.8	Random sampling	cross-sectional survey
Caoxin, Bao et al., 2015	5 tertiary hospitals	2170 outpatient	2170 outpatients Male: 870 Female: 1300	Random sampling	cross-sectional survey
Zhixiang, Teng et al., 2009	Residents survey	134 residents who visited the hospitals in the last year, of whom, 40 visited the tertiary hospital	NR	Convenient sampling	cross-sectional survey
Jing Luan et al., 2013	Residents survey	510 residents	Male: 178 Female: 332	Random sampling	cross-sectional survey
Jianjie, Zhang. 2018	Residents survey	484 residents	NR	Random sampling	cross-sectional survey
Chunhui, Ren. 2014	Residents Survey	329 participants	NR	NR	cross-sectional survey
Weiming, Shao et al., 2017	A tertiary hospital	600 outpatients	NR	Random sampling	cross-sectional survey
Ying Zou et al., 2014	A Tertiary hospital in Xiniiang Province	1300 outpatients	NR	Random sampling	cross-sectional survey
Mengzhu, Deng et al., 2013	A Tertiary hospital in Guangdong Province	200 outpatients	Male: 110 Female: 90	Random sampling	cross-sectional survey
Huilan, Luo et al., 2010	A tertiary hospital in Guangdong Province	1591 outpatients	NR	Random sampling	cross-sectional survey
Zulipiye.et al., 2016	A tertiary hospital in Xinjiang Province	1300 outpatients	Female: 736 Male: 564 Age range: 18–69 y	Random sampling	cross-sectional survey
Jinping, Shu and Dian Zhou. 2016	A tertiary hospital in Anhui Province	396 outpatients	Male: 171 Female: 225	Stratified cluster sampling	cross-sectional survey
Kai ling et al., 2009	A tertiary hospital	500 outpatients	Male: 307 Female: 193 Aged range: 60–85	NR	cross-sectional survey
Yu Li et al., 2011	Five tertiary Hospitals in Tianjian City	995 outpatients	Male: 438 Female: 557 Aged range: 20–70	Random sampling	cross-sectional survey
Qinghua, Zhang and Zhanhe, Liu. 2011	A tertiary hospital in Hebei Province	817 outpatients	NR	Random sampling	cross-sectional survey
Fanzhi, Meng et al., 2016	Residents survey covering 2 cities, 3 counties and 5 villages in Tibet province	777 residents	Male: 369 Female: 408 Age range: 18–40	Stratified clustered random sampling	cross-sectional survey
Jun Song et al., 2007	A tertiary hospital in Jiangsu Province	93 outpatients	Male: 49 Female: 44 Mean age: 37.6 ± 4.3 y	Random sampling	cross-sectional survey
Shi Guo et al., 2014	A tertiary hospital in Anhui Province	239 outpatients	Male: 118 Female: 121	Stratified random sampling	cross-sectional survey
Xunming, Ji et al., 2010	A tertiary hospital in Beijing	504 outpatients	Media age: 47	Convenient sampling	cross-sectional survey
Li Ren and Haixuan, Xu. 2016	A tertiary hospital in Shandong Province	488 outpatients	Male: 216 Female: 272	Random sampling	cross-sectional survey
Guanghao, Jing and Shunfu, Piao. 2011	A tertiary hospital	597 outpatients	NR	NR	cross-sectional survey
Lizhen, Deng and Suili, Rao. 2006	A tertiary hospital	1226 outpatients	NR	Random sampling	cross-sectional survey

* NR means not reported. † This study was reported by two papers. Titles of each paper were listed in Supplementary Material 4.

**Table 2:** Quality of included studies (*n* = 35).

Author and Year	Sample Selection Criteria (Maximum of 4 Stars)				Comparability (Maximum 2 Stars)	Outcome (Maximum 1 Star)	Summary Score (Maximum of 7 Stars)
	Representativeness of the Sample	Sample Size	Non-Respondents	Ascertainment of the Satisfaction Level	Comparability of Subjects in Different Outcome Groups; Control of Confounding Factors.	Assessment of the Outcome from Patient’s Point of View	
Wenya, Yu et al., 2016. Shanghai	Truly representative of the average in the target population *	Justified and satisfactory *	Comparability between respondents’ and nonrespondents’ characteristics is established, and the response rate is satisfactory *	Validated measurement tool *	The study controls for the most important factor **;	Self-report *	7
Liyang Tang, 2011	Somewhat representative of the average in the target population *	Justified and satisfactory *	Comparability between respondents’ and nonrespondents’ characteristics is established, and the response rate is satisfactory *	Non validated measurement tool, but the tool is available or described *;	The study controls for the most important factor **;	Self-report *	7
Jing Sun, et al., 2017 Laiyang Wu, et al., 2016	Truly representative of the average in the target population *	Justified and satisfactory *	No description	Non validated measurement tool, but the tool is available or described *;	The study controls for the most important factor **;	Self-report *	6
Jay Pan, et al., 2015	Truly representative of the average in the target population *	Justified and satisfactory *	Comparability between respondents’ and nonrespondents’ characteristics is established, and the response rate is satisfactory *	Validated measurement tool *	The study controls for the most important factor **;	Self-report *	7
Jinghua Li et al., 2016	Truly representative of the average in the target population *	Justified and satisfactory *	Comparability between respondents’ and nonrespondents’ characteristics is established, and the response rate is satisfactory *	Non validated measurement tool, but the tool is available or described *;	The study controls for the most important factor **;	Self-report *	7
Jinzhu Xie, et al., 2017	Somewhat representative of the average in the target population *	Not justified	Comparability between respondents’ and nonrespondents’ characteristics is established, and the response rate is satisfactory *	Non validated measurement tool, but the tool is available or described *;	The study controls for the most important factor **;	Self-report *	6
Chunlei Han et al., 2000	Truly representative of the average in the target population *	Not justified	Comparability between respondents’ and nonrespondents’ characteristics is established, and the response rate is satisfactory *	Non validated measurement tool, but the tool is available or described *;	data not adjusted for all relevant confounders	Self-report *	4
Qing Lu et al., 2016	Truly representative of the average in the target population *	Not justified	Comparability between respondents’ and nonrespondents’ characteristics is established, and the response rate is satisfactory *	Non validated measurement tool, but the tool is available or described *;	NA	Self-report *	4
Jing Zhao et al., 2015	Truly representative of the average in the target population *	Not justified	Comparability between respondents’ and nonrespondents’ characteristics is established, and the response rate is satisfactory *	Non validated measurement tool, but the tool is available or described *;	NA	Self-report *	4
Wenlong Hu et al., 2007	Truly representative of the average in the target population *	Not justified	Comparability between respondents’ and nonrespondents’ characteristics is established, and the response rate is satisfactory *	Non validated measurement tool, but the tool is available or described *;	NA	Self-report *	4
Yanxia Yang et al., 2015	Truly representative of the average in the target population *	Not justified	Comparability between respondents’ and nonrespondents’ characteristics is established, and the response rate is satisfactory *	Non validated measurement tool, but the tool is available or described *;	data not adjusted for all relevant confounders	Self-report *	4
Rong Xu and Xinzhen Jing. 2004	Somewhat representative of the average in the target population *	Not justified	No description	Validated measurement tool *	NA	Self-report *	3
Junjie Sun and Shuangqing Li, 2018	Somewhat representative of the average in the target population *	Not justified	Comparability between respondents’ and nonrespondents’ characteristics is established, and the response rate is satisfactory *	Validated measurement tool *	data not adjusted for all relevant confounders	Self-report *	4
Zhanwei Zhou, et al., 2011	Truly representative of the average in the target population *	Justified and satisfactory *	Comparability between respondents’ and nonrespondents’ characteristics is established, and the response rate is satisfactory *	No description	data not adjusted for all relevant confounders	Self-report *	3
Caoxin, Bao, et al., 2015	Truly representative of the average in the target population *	Justified and satisfactory *	No description	Validated measurement tool *	NA	Self-report *	4
Zhixiang, Teng, et al., 2009	Somewhat representative of the average in the target population *	Not justified	Response rate is unsatisfactory, or the comparability between respondents and nonrespondens is unsatisfactory;	Validated measurement tool *	NA	Self-report *	3
Jing Luan, et al., 2013	Truly representative of the average in the target population *	Justified and satisfactory *	Comparability between respondents’ and nonrespondents’ characteristics is established, and the response rate is satisfactory *	Validated measurement tool *	NA	Self-report *	5
Jianjie Zhang. 2018	Truly representative of the average in the target population *	Not justified	Comparability between respondents’ and nonrespondents’ characteristics is established, and the response rate is satisfactory *	Validated measurement tool *	The study controls for the most important factor **;	Self-report *	6
Chunhui, Ren. 2014	Selected group of patients	Not justified	Comparability between respondents’ and nonrespondents’ characteristics is established, and the response rate is satisfactory *	Validated measurement tool *	NA	Self-report *	3
Weiming Shao et al., 2017	Truly representative of the average in the target population *	Not justified	Comparability between respondents’ and nonrespondents’ characteristics is established, and the response rate is satisfactory *	Non validated measurement tool, but the tool is available or described *;	NA	Self-report *	4
Ying Zou et al., 2014	Truly representative of the average in the target population *	Not justified	Comparability between respondents’ and nonrespondents’ characteristics is established, and the response rate is satisfactory *	Validated measurement tool *	NA	Independent blind assessment *	4
Mengzhu Deng et al., 2013	Somewhat representative of the average in the target population *	Not justified	Comparability between respondents’ and nonrespondents’ characteristics is established, and the response rate is satisfactory *	Validated measurement tool *	data not adjusted for all relevant confounders	Self-report *	4
Huilan Luo et al., 2010	Truly representative of the average in the target population *	Not justified	Comparability between respondents’ and nonrespondents’ characteristics is established, and the response rate is satisfactory *	Validated measurement tool *	NA	Self-report *	4
Zulipiye Tuerxun et al., 2016	Truly representative of the average in the target population *	Not justified	Comparability between respondents’ and nonrespondents’ characteristics is established, and the response rate is satisfactory *	Non validated measurement tool, but the tool is available or described *;	NA	Self-report *	4
Jinping Shu and Dian Zhou, 2016	Truly representative of the average in the target population *	Not justified	Comparability between respondents’ and nonrespondents’ characteristics is established, and the response rate is satisfactory *	Validated measurement tool *	The study controls for the most important factor **;	Self-report *	6
Kai Ling et al., 2009	Somewhat representative of the average in the target population *	Not justified	Comparability between respondents’ and nonrespondents’ characteristics is established, and the response rate is satisfactory *	Non validated measurement tool, but the tool is available or described *;	NA	No description.	3
Yu Li, et al., 2011	Truly representative of the average in the target population *	Not justified	Comparability between respondents’ and nonrespondents’ characteristics is established, and the response rate is satisfactory *	Non validated measurement tool, but the tool is available or described *;	NA	Self-report *	4
Qinghua Zhang and Zhanhe Liu. 2011	Truly representative of the average in the target population *	Not justified	Comparability between respondents’ and nonrespondents’ characteristics is established, and the response rate is satisfactory *	Non validated measurement tool, but the tool is available or described *;	NA	Self-report *	4
Zhifan Meng, et al., 2016	Truly representative of the average in the target population *	Not justified	Comparability between respondents’ and nonrespondents’ characteristics is established, and the response rate is satisfactory *	Non validated measurement tool, but the tool is available or described *;	NA	Self-report *	4
Jun Song, et al., 2007	Truly representative of the average in the target population *	Not justified	Comparability between respondents’ and nonrespondents’ characteristics is established, and the response rate is satisfactory *	Non validated measurement tool, but the tool is available or described *;	NA	Self-report *	4
Shi Guo, et al., 2014	Truly representative of the average in the target population *	Not justified	Comparability between respondents’ and nonrespondents’ characteristics is established, and the response rate is satisfactory *	Non validated measurement tool, but the tool is available or described *;	NA	Self-report *	4
Xunming Ji, et al., 2010	Somewhat representative of the average in the target population *	Not justified	No description	Non validated measurement tool, but the tool is available or described *;	NA	Self-report *	3
Li Ren and Haixuan Xu.2016	Truly representative of the average in the target population *	Not justified	No description	Non validated measurement tool, but the tool is available or described *;	NA	Self-report *	3
Guanghao Jing and Shunfu Piao. 2011	Truly representative of the average in the target population *	Not justified	Comparability between respondents’ and nonrespondents’ characteristics is established, and the response rate is satisfactory *	Non validated measurement tool, but the tool is available or described *;	NA	Self-report *	4
Lizhen, Deng and Suili, Rao 2006	Truly representative of the average in the target population *	Not justified	Comparability between respondents’ and nonrespondents’ characteristics is established, and the response rate is satisfactory *	Non validated measurement tool, but the tool is available or described *;	NA	Self-report *	4

* the detailed explanation of the number of the stars were in Supplementary Material 2. NA means not applicable and represented that the study did not investigated the factors influencing the outpatient satisfaction.

**Table 3:** Findings on levels of outpatient satisfaction of included studies (*n* = 35).

Author and Year	Assessment Instrument	Response Rate (%)	Findings on Levels of Outpatient Satisfaction
Wenya, Yu et al., 2016	Adapted questionnaire from the IPSQ ^‡^ (A 5-point Likert scale questionnaire)	92.48	The mean overall outpatient satisfaction was 4.0 ± 0.7. Satisfaction with service attitude was the highest, while satisfaction with medical needs being met by doctors was the lowest.
Liyang, Tang. 2011	A self-designed 5-point Likert scale questionnaire	100	The mean overall outpatient satisfaction was 3.7 ± 0.76 Satisfaction with doctor-patient interaction was the highest, while satisfaction with waiting time in hospital was the lowest.
Jing Sun et al., Laiyang, Wu et al., 2016	A self-designed 5-point Likert scale questionnaire.	NR	The overall satisfaction score is 4.42 ± 0.68 Satisfaction with diagnosis and treatment was the highest, while satisfaction with long waiting time in hospital was the lowest
Jay Pan et al., 2015	A self-designed 5-point Likert scale questionnaire	100%	Satisfaction with medical charges was the lowest.
Jinghua, Li et al., 2016	A self-designed questionnaire (mixed up with 3-point and 5-point Likert scale)	NR	The satisfaction of outpatients from county and tertiary hospitals were significantly lower than those visited village/township clinics.
Jinzhu, Xie et al., 2017	A self-designed 5-point Likert scale questionnaire.	97.2	Satisfaction with information got was the highest, while satisfaction with medical cost was the lowest
Chunlei, Han et al., 2012	A self-designed questionnaire based on IPSQ	99.4	Satisfaction with hospital management is high, while satisfaction with waiting time is low.
Qing Lu et al., 2016	A self-designed five-point Likert scale questionnaire	90.9	82. 70% (263 /318) of the study participants stated general satisfaction with the outpatient.
Jing Zhao et al., 2016	A self-designed five-point Likert scale questionnaire	98.5	Satisfaction with professional skills of doctors was highest, with outpatient management is lowest.
Wenlong, Hu et al., 2007	NR	96	The overall satisfaction to the outpatient medical service is 76.9%. The top 3 factors outpatients mostly satisfied with are doctors’ professionalism, hospital hygiene and clear diagnosis. The top 3 factors outpatients mostly dissatisfied with are high medical costing, long waiting time and complicated formalities.
Yanxia, Yang et al., 2015	A self-designed questionnaire	99.42	Waiting time, personal health conditions, and knowledge about the diseases are important factors related to outpatient satisfaction.
Rong Xu and Xinzhen, Jing. 2004	A self-designed five-point Likert scale questionnaire	98.7	Satisfaction with hospital hygiene was highest, and satisfaction with waiting time of medical examinations was lowest.
Junjie, Sun and Shuangqing, Li. 2018	A self-designed five-point Likert scale questionnaire	92.5	The satisfaction of the outpatients is 87.6% (162/185)
Zhanwei, Zhou et al., 2011	A self-designed questionnaire	97.4	The outpatients generally showed a high degree of satisfaction with doctors and a low satisfaction with the service staff (Hospital billing collector)
Caoxin, Bao et al., 2015	A self-designed five-point Likert scale questionnaire	NR	The outpatients’ satisfaction score is 82.48. Satisfaction with the overall evaluation of the work and the medical staff was highest; atisfaction with the treatment effect, cost, and the administrative staff was lowest
Zhixiang, Teng et al., 2009	A self-designed seven-point Likert scale questionnaire	68.7	The outpatient satisfaction score is 4.23 ± 1.14.
Jing Luan et al., 2013	A self-designed five-point Likert scale questionnaire	92.8	NR *
Jianjie Zhang. 2018	A self-designed seven-point Likert scale questionnaire	90.1	NR
Chunhui, Ren. 2014	A self-designed questionnaire	98	The outpatients were mostly satisfied with the service facilities, followed by medical equipment, and hygiene et al., and mostly dissatisfied with service attitude and medical costs.
Weiming, Shao et al., 2017	A self-designed five-point Likert scale questionnaire	100	The outpatients were mostly satisfied with clean hospital hygiene, and mostly dissatisfied with long waiting time.
Ying Zou et al., 2014	A self-designed questionnaire	99	The outpatients were mostly satisfied with the hospital hygiene, and mostly dissatisfied with the arrangement of the outpatient department, long waiting time, and shortage of expert outpatients.
Mengzhu, Deng et al., 2013	A self-designed questionnaire	96.15	The outpatients were mostly satisfied with the service attitude and environmental facility, and mostly dissatisfied with the waiting time and medical expenses.
Huilan, Luo et al., 2010	A self-designed three- point Likert scale questionnaire based on IPSQ	99	The outpatients were mostly dissatisfied with long waiting time, hospital hygiene and outpatient procedures guidance.
Zulipiye.et al., 2016	A self-designed five- point Likert scale questionnaire	100	Outpatients were mostly satisfied with hospital hygiene and medical service, and were mostly dissatisfied with long waiting time and complicated formalities during outpatients.
Jinping, Shu and Dian Zhou. 2016	A self-designed five-point Likert scale questionnaire	94	Outpatients were mostly dissatisfied with the outpatient process, including the registration and taking drugs et al.,
Kai ling et al., 2009	A self-designed four-point Likert scale questionnaire	100	Senior outpatients were mostly dissatisfied with the long waiting time.
Yu Li et al., 2011	A self-designed five-point Likert scale questionnaire	99.5	The outpatients were mostly satisfied with the doctor service attitude and environmental, and were mostly dissatisfied with the treatment time and health care costs.
Qinghua, Zhang and Zhanhe, Liu. 2011	A self-designed three-point Likert scale questionnaire	91	The outpatients were mostly dissatisfied with charge offices and pharmacy.
Fanzhi, Meng et al., 2016	NR	100	94.6% residents showed satisfaction with the outpatient. The residents were mostly dissatisfied with low accessibility to various of drugs.
Jun Song et al., 2007	A self-designed four-point Likert scale questionnaire	93	The patients were mostly satisfied with the examination time and guidance during outpatient.
Shi Guo et al., 2014	A self-designed five-point Likert scale questionnaire	97.5	Patients were mostly satisfied with the medical diagnosis and treatment, and were mostly dissatisfied with waiting time before seeing the doctors and medical costs.
Xunming, Ji et al., 2010	A self-designed five-point Likert scale questionnaire	98.82	86% outpatients were satisfied with the medical care at outpatients’ departments, and were mostly dissatisfied with professional skills, service attitude and time consuming.
Li Ren and Haixuan, Xu. 2016	A self-designed five-point Likert scale questionnaire	97.6	The outpatients were mostly satisfied with examination time of the doctors, and mostly dissatisfied with waiting time for the examination and reports.
Guanghao, Jing and Shunfu, Piao. 2011	A self-designed three-point Likert scale questionnaire	99.5	90.3% outpatients were satisfied with the outpatient. The outpatients were mostly dissatisfied with complicated outpatient process, cold service attitude, unclear explanation, and long waiting time.
Lizhen, Deng and Suili, Rao. 2006	A self-designed questionnaire based on the outpatient questionnaire designed by the ministry of health Guangzhou city	85	Outpatients were mostly satisfied with no corruption and good service attitude of the doctors and nurses. Outpatients were mostly dissatisfied with the service attitude of registration and payment offices.

^‡^ IPSQ: Inpatient Satisfaction Questionnaire; * NR means not reported.

**Table 4:** Satisfaction with hospital staff, hospital hygiene and facilities, outpatient process and management, and medical costs (*n* = 22).

Studies	Satisfaction with Hospital Staff					Satisfaction with Hospital Indoor Hygiene and Facilities (The Arrangement, Clear Instruction, Signs, Hygiene, and Enough Seats)	Satisfaction with Outpatient Process and Management					Satisfaction with Medical Costs
Author and Year	Service Attitude or Communication with the Doctors	Service Attitude or Communication with the Nurses	Professional Skills	Service of the Guidance Medical Staff/Pre-Diagnosis Counters	Service Attitude of the Registration/Payment Offices		Easy Access to Hospital and Registration	Waiting Time	Diagnosis and Treatment Process	Ease of Complain (a Clear and Reliable Channel for Praise and Complain)	Medical Needs Being Met	
Wenya, Yu et al., 2016	*	*	*	*		*		*			*	*
Jing Sun et al., 2015 Laiyang wu, et al., 2016	*	*	*			*	*	*	*	*		
Weiming Shao et al., 2017	*			*	*	*		*	*	*		*
Ying Zou et al., 2014		*		*	*	*			*	*		
Mengzhu Deng et al., 2013	*	*				*		*				*
Qing Lu et al., 2016	*	*	*		*	*		*				*
Jing Zhao et al., 2015	*	*	*			*	*					
Huilan Luo et al., 2010								*	*			*
Zulipiye Tuerxun et al., 2016	*	*	*	*		*	*	*	*			
Jinping Shu and Dian Zhou, 2016	*		*	*	*	*	*	*	*			*
Kai Ling et al., 2009		*	*			*						
Yu Li, et al., 2011	*	*	*	*	*	*		*	*			*
Rong Xu and Xinzhen Jing. 2004	*	*	*	*	*			*			*	*
Qinghua Zhang and Zhanhe Liu. 2011	*	*	*	*	*	*			*			
Zhifan Meng, et al., 2016	*	*				*		*				*
Jun Song et al., 2007	*	*	*					*	*			*
Shi Guo, et al., 2014	*	*				*		*	*		*	
Xunming Ji, et al., 2010	*		*					*				
Li Ren and Haixuan Xu. 2016	*	*		*	*	*		*				
Guanghao Jing and Shunfu Piao. 2011	*	*	*			*			*			*
Lizhen, Deng and Suili, Rao 2006	*	*	*		*	*			*		*	*
Chunhui, Ren. 2014	*	*				*	*					*

* An asterisk mark represented that this factor was investigated in the study. Yellow color represented the most satisfied factors, and blue color represented the most dissatisfied factor.

**Table 5:** Findings on factors associated with outpatient satisfaction (*n* = 18).

Author and Year	Main Findings on the Relationship between Outpatient Satisfaction and Influencing Factors		
	Patient Social-Demographic Factors	Medial Staff Factors	hygiene and Process Management
Wenya, Yu et al., 2016	(1) Outpatients’ socio-demographic characteristics (including sex, age, occupation, monthly income, residence, and marital status) were related to satisfaction to varying degrees. (2) Outpatients who were male, older, married, with low or middle incomes (2000–4999 Yuan), living in Shanghai, or students were more satisfied than those without these characteristics. (3) Young and middle-aged adults (20–39 years), and divorced or widowed patients had lower odds of high satisfaction with doctors.	Satisfactions with doctors and with nurses were significantly related to the overall satisfaction.	Satisfaction with hygiene had the weakest contribution to overall satisfaction.
Liyang, Tang. 2011	Patients’ trust in medical service had the largest influence on patient’s satisfaction.	NR *	NR
Jing Sun et al., 2017 Laiyang, Wu et al., 2016	(1) Outpatient with commercial insurance coverage is associated with satisfaction that is 1.73 times that of the uninsured (*p* = 0.03) (2) Satisfaction scores of the Chinese elderly outpatients were significantly higher than that of the young and middle-aged outpatients in the domains of hospital hygiene, process efficiency, and overall satisfaction (*p* < 0.001). On the contrary, the elderly outpatients were less satisfied in the domain of hospital informationization experience than the young and middle-aged outpatients.	(1) “Patient–doctor relationship” is the strongest predictor of overall patient satisfaction (OR = 3.19, 95% CI: 2.83–3.59); (2) Trustful doctor–patient relationship (OR = 3.45), respected and comfortable care (OR = 1.45), clear and reliable mechanism, length of communication time with doctors (OR = 1.35). and waiting time (OR = 1.29) were major factors associated with the overall satisfaction of the elderly outpatients	(1) Channel for praise and complain (OR = 1.39) was the major factors associated with the overall satisfaction of the elderly outpatient. (2) Hospital hygiene, process management, and healthcare experience significantly correlated with outpatient satisfaction;
Jay Pan et al., 2015	(1) Female are less dissatisfied; (2) Higher income is associated with lower satisfaction level in outpatient satisfaction level;	NR	NR
Jinghua, Li et al., 2016	(1) Men and singles were less likely to be satisfied with waiting time. (2) Individuals aged 15–44 years were less likely to be satisfied compared with those aged ≥65 years. (3) Higher education was associated with lower odds of satisfaction. (4) Employed individuals were much more likely to be satisfied. (5) People living in urban areas were less likely to report satisfaction than people living in rural areas (6) Insured patients were much more likely to be satisfied compared with uninsured patients	NR.	(1) Patients seeking outpatient care from tertiary hospitals were very satisfied with the care environment, whereas those in rural areas were less satisfied (*p* = 0.008); (2) Among patients seeking outpatient care from tertiary hospitals, the odds of satisfaction with waiting time and medical costs were significantly lower than those using village/township clinics.
Jinzhu, Xie et al., 2017	Age, type of payment, and the self-rated health status were associated with outpatient satisfaction; Outpatients older than 65 years had the highest experience score, whereas outpatients paying out-of-pocket had the lowest experience score.	NR	NR
Chunlei, Han et al., 2012	Patients’ demographic characteristics including occupation, monthly salary, and education level were associated with outpatient satisfaction.	NR	NR
Qing Lu et al., 2016	Marital status, occupation, health insurance type, payment-method, and family income were correlated with outpatient satisfaction.	NR	NR
Jing Zhao et al., 2016	NR	NR	Outpatient process was an independent factor influencing outpatient satisfaction.
Wenlong, Hu et al., 2007	Age, gender and monthly income are not significantly associated with outpatient satisfaction, while level of education was significantly associated with outpatient satisfaction.	NR	NR
Yanxia, Yang et al., 2015	There were no significant differences in patients’ sex, age, marriage, occupation and education on outpatient satisfaction.	NR	outpatient satisfaction was associated with waiting time.
Rong Xu and Xinzhen, Jing. 2004	NR	The outpatient satisfaction was mostly associated with the satisfaction with the diagnosis and treatment of the doctor, and the service attitude of the doctors.	The outpatient satisfaction was mostly associated with the satisfaction with the medical cost and the arrangements during the wait.
Junjie, Sun and Shuangqing, Li. 2018	NR	Communication with doctors, the degree of carefulness the doctors inquired, and the degree of the clarity the doctors explained the diseases are associated with outpatient satisfaction.	NR
Zhanwei, Zhou et al., 2011	NR	Patients showed significant lower satisfaction with payment officers compared with doctors and nurses. The feeling of being respect during outpatient, professional skills of doctors and the service of the pre-diagnosis counters were associated with outpatient satisfaction.	Hospital hygiene and medical costs are associated with outpatient satisfaction.
Caoxin, Bao et al., 2015	NR	The service attitude and professional skills of doctors are associated with outpatient satisfaction	Hospital reputation, the protection of privacy during diagnosis and waiting time are associated with outpatient satisfaction.
Zhixiang, Teng et al., 2009	NR	NR	The levels of the hospitals are not associated with outpatient satisfaction in the first-time visit.
Jing Luan et al., 2013	The age, gender, marital status, education level, monthly income and self-health evaluation are associated with outpatient satisfaction.	NR	NR
Jianjie Zhang. 2018	Health-related knowledge is positively associated with outpatient satisfaction.	The professional skills were of significantly positive correlated with outpatient satisfaction.	Waiting time is not associated with outpatient satisfaction.

* NR means not reported.

## Supplementary Materials

The following are available online at https://www.mdpi.com/1660-4601/17/19/7070/s1. Supplementary material 1: Databases searched and search strategy, Supplementary material 2: Tool used for quality assessment: NEWCASTLE—OTTAWA QUALITY ASSESSMENT SCALE (adapted for cross sectional studies), Supplementary material 3: Papers excluded after reading full texts with the reasons (*n* = 190), Supplementary material 4: Titles of include studies.
