# Supplementary material for: Factors Associated with Outpatient Satisfaction in Tertiary Hospitals in China: A Systematic Review
Source: Int J Environ Res Public Health. 2020 Sep 27;17(19):7070. doi: 10.3390/ijerph17197070 (PMC7579147; doi:10.3390/ijerph17197070)
Supplement: Supplementary file 1 [file ijerph-17-07070-s001.pdf]

Supplementary Material 1. Databases searched and search strategy

Database	Search strings and search strategy	Searching results
Cochrane<br>http://www.cochranelibrary.com	'"china" in Title, Abstract, Keywords and "patient" in Title, Abstract, Keywords and "satisfaction" in Title, Abstract, Keywords in Cochrane Reviews	12
Cochrane Central Register of Controlled Trials<br>http://www.cochranelibrary.com	"china" in Title, Abstract, Keywords and "patient" in Title, Abstract, Keywords and "satisfaction" in Title, Abstract, Keywords in Trials'	187
PubMed<br>http://www.ncbi.nln.nih.gov/pubmed	("Patient Satisfaction"[MeSH Terms] OR ("patient"[All Fields] AND "satisfaction"[All Fields]) OR "Patient Satisfaction"[All Fields] OR "Patient Satisfaction"[MeSH Terms]) AND ("China"[MeSH Terms] OR "China"[All Fields] OR "china s"[All Fields] OR "chinas"[All Fields] OR "China"[MeSH Terms]) AND 1946/01/01:2018/02/28[Date - Publication]	2577
Web of Science<br>http://apps.webofknowledge.com	TOPIC: (Patient satisfaction) AND TOPIC: (China OR Chinese) Timespan: All years. Indexes: SCI-EXPANDED, SSCI.	891
EMBASE (Science direct)<br>http://embase.com/search	(Patient satisfaction) AND (China OR Chinese)	3064
China National Knowledge Infrastructure<br>http://www.cnki.net	(Hospitalize OR go to a doctor) AND (satisfaction OR experience)	574
CQVIP<br>www.cqvip.com	(Hospitalize OR go to a doctor) AND (satisfaction OR experience)	502

Supplementary Material 2: Tool used for quality assessment: NEWCASTLE - OTTAWA QUALITY ASSESSMENT SCALE (adapted for cross sectional studies)

Selection: (Maximum 5 stars)

1) Representativeness of the sample:

a) Truly representative of the average in the target population. * (all subjects or random sampling)

b) Somewhat representative of the average in the target population. * (non-random sampling)

c) Selected group of users.

d) No description of the sampling strategy.

2) Sample size:

a) Justified and satisfactory. *

b) Not justified. 3) Non-respondents:

a) Comparability between respondents and non-respondents characteristics is established, and the response rate is satisfactory. *

b) The response rate is unsatisfactory, or the comparability between respondents and non-respondents is unsatisfactory.

c) No description of the response rate or the characteristics of the responders and the non-responders.

4) Ascertainment of the exposure (risk factor):

a) Validated measurement tool. **

b) Non-validated measurement tool, but the tool is available or described.*

c) No description of the measurement tool.

Comparability: (Maximum 2 stars)

1) The subjects in different outcome groups are comparable, based on the study design or analysis. Confounding factors are controlled.

a) The study controls for the most important factor (select one). * b) The study control for any additional factor. *

Outcome: (Maximum 3 stars)

1) Assessment of the outcome:

a) Independent blind assessment. ** b) Record linkage. **c) Self report. *d) No description.

2) Statistical test:

a) The statistical test used to analyze the data is clearly described and appropriate, and the measurement of the association is presented, including
confidence intervals and the probability level (p value). *

b) The statistical test is not appropriate, not described or incomplete.

Supplementary Material 3. Papers excluded after reading full texts with the reasons (n=190):

The numbers represented the reasons for exclusion:

1: Participants were not Chinese or studies taken place outside of China;
2: Studies investigated the satisfaction with inpatient, with nursing, with health education, or with primary health care and surgery et al;
3: Evaluation of the effectiveness of specific interventions/program/treatment on improving outpatient or inpatient satisfaction;
4: Studies investigated the satisfaction of medical staff or hospital managers with outpatient or inpatient;
5: Studies validated patient satisfaction investigation tools;
6: Studies investigated satisfaction within specific department;
7: Studies not taken place in tertiary hospital (e.g., community health centers);
8: The factors associated with the outpatient satisfaction or was not investigated, only satisfaction rate is reported;
9: Reviews

Title of papers excluded	Exclusion of reasons
Hypertension Prevalence, Health Service Utilization, and Participant Satisfaction: findings From a Pilot Randomized Controlled Trial in Aged Chinese Canadians	1
Telephone follow-up for patients returning home with colostomies: views and experiences of patients and enterostomal nurses	3
Questionnaire survey about use of an online appointment booking system in one large tertiary public hospital outpatient service centre in China	3
An evaluation of activity tolerance, patient-reported outcomes and satisfaction with the effectiveness of pulmonary daoyin on patients with chronic obstructive pulmonary disease	3
Effects of a health-social partnership transitional program on hospital readmission: a randomized controlled trial	3
Patient satisfaction after nurse-led care in Chinese patients with rheumatoid arthritis: a China study	3
Continuous primary nursing care increases satisfaction with nursing care and reduces postpartum problems for hospitalized pregnant women	3
Feedback intervention to doctors improves patient satisfaction among outpatients in Inner Mongolia Autonomous Region, China	3
Factors related to client satisfaction with methadone maintenance treatment in China	3
The effect of integrated health management model on the satisfaction among Chinese elderly	3
Utilization, cost, payment, and patient satisfaction of rehabilitative services in Shandong, China	3
Unhappy Patients Are Not Alike: Content Analysis of the Negative Comments from China's Good Doctor Website	3
The relation between patient education, patient empowerment and patient satisfaction: A cross-sectional-comparison study	2
Treatment satisfaction with paliperidone extended-release tablets: open-label study in schizophrenia patients dissatisfied with previous antipsychotic medication	3

Patient satisfaction and gender composition of physicians - a cross-sectional study of community health services in Hubei, China	7
Patient Satisfaction with Community Health Service Centers as Gatekeepers and the Influencing Factors: A Cross-Sectional Study in Shenzhen, China	7
Service provision, pricing, and patient satisfaction in online health communities	3
Health system reforms, violence against doctors and job satisfaction in the medical profession: a cross-sectional survey in Zhejiang Province, Eastern China	4
Development of an In-Patient Satisfaction Questionnaire for the Chinese Population	2
Chinese patients' satisfaction with total hip arthroplasty: what is important and dissatisfactory?	2
Patient satisfaction with fixed-combination bimatoprost/timolol ophthalmic solution: a survey study in patients with glaucoma in China	2
Reducing waiting time and raising outpatient satisfaction in a Chinese public tertiary general hospital-an interrupted time series study	3
Validation of a Measure of Chinese Outpatients' Satisfaction in the Taiwan Setting	5
The Impact of Ethnic and Cultural Differences on Parental Satisfaction in the PICU	1
The frequency of patient-initiated violence and its psychological impact on physicians in china: a cross-sectional study	4
Nurse employment contracts in Chinese hospitals: impact of inequitable benefit structures on nurse and patient satisfaction	2
Patient Satisfaction with Hospital Inpatient Care: Effects of Trust, Medical Insurance and Perceived Quality of Care	2
Perceptions, knowledge, and satisfaction with contralateral prophylactic mastectomy among young women with breast cancer: a cross-sectional survey	1
Patient satisfaction with prescribed medicines in community health services in China: A cross-sectional survey 6 years after the implementation of the national essential medicines policy	7
Characterization of patients and its relationship with medical complaints	2
Patient satisfaction with primary care: a comparison between conventional care and traditional Chinese medicine	1
Doctor-patient relationships (DPR) in China	4
A satisfaction survey of current medicines used for migraine therapy in China: is Chinese patent medicine effective compared with Western medicine for the acute treatment of migraine?	3
Nurse burnout in China: a questionnaire survey on staffing, job satisfaction, and quality of care	4
[Using satisfaction questionnaire status version (DTSQs) to study on the reliability, construct validity and responsiveness of diabetes treatment among Chinese diabetic outpatients]	5

Assessing patient satisfaction with medication related services in hospital settings: a cross-sectional questionnaire survey in China	2
Clozapine in schizophrenia and its association with treatment satisfaction and quality of life: Findings of the three national surveys on use of psychotropic medications in China (2002-2012)	2
Antipsychotic polypharmacy in schizophrenia patients in China and its association with treatment satisfaction and quality of life: findings of the third national survey on use of psychotropic medications in China	2
Evaluation of medical staff and patient satisfaction of Chinese hospitals and measures for improvement	2
Patient satisfaction between primary care providers and hospitals: a cross-sectional survey in Jilin province, China	2
Patient satisfaction after ambulatory inguinal hernia repair in Hong Kong	2
Factors predicting patient satisfaction in women with advanced breast cancer: a prospective study	2
Does the use of shared decision-making consultation behaviors increase treatment decision-making satisfaction among Chinese women facing decision for breast cancer surgery?	3
Patient and family satisfaction levels in the intensive care unit after elective cardiac surgery: study protocol for a randomised controlled trial of a preoperative patient education intervention	3
A descriptive quantitative study on multi-ethnic patient satisfaction with nursing care measured by the Revised Humane Caring Scale	1
Patient satisfaction after posterior-stabilized total knee arthroplasty: a functional specific analysis	2
Residents' satisfaction with primary medical and health services in Western China	2
Maintenance inhaler preference, attribute importance, and satisfaction in prescribing physicians and patients with asthma, COPD, or asthma-COPD overlap syndrome consulting for routine care	2
Satisfaction among early and mid-career dentists in a metropolitan dental hospital in China	4
Clocks Moving at Different Speeds: Cultural Variation in the Satisfaction With Wait Time for Outpatient Care	1
Factors influencing inpatients' satisfaction with hospitalization service in public hospitals in Shanghai, People's Republic of China	2
Factors that influence in vitro fertilization treatment outcomes of Chinese men: A cross-sectional study	2
Impact of adjustment measures on reducing outpatient waiting time in a community hospital: application of a computer simulation	3
Satisfaction with inpatient care in a population-based Hong Kong Chinese sample	2
A web-based appointment system to reduce waiting for outpatients: a retrospective study	3
Hospital organizational environment and staff satisfaction in China: A large-scale survey	5

Interpretation of illness in patients with chronic diseases from Shanghai and their associations with life satisfaction, escape from illness, and ability to reflect the implications of illness	2
Enhancing outpatient clinics management software by reducing patients' waiting time	1
Improving Efficiency and Patient Satisfaction in a Peripherally Inserted Central Catheter Center Using Lean-Based Methodology	3
Exploring the influence of the online physician service delivery process on patient satisfaction	2
Evaluation of patients' satisfaction, need, and perceived barriers in cancer care at Tianjin Cancer Institute Hospital	2
Healthcare in the New Vietnam: Comparing patients' satisfaction with outpatient care in a traditional neighborhood clinic and a new, western-style clinic in Ho Chi Minh City	1
Use of patient satisfaction data in a continuous quality improvement program for endoscopic sinus surgery	3
Patient–physicians' information exchange in outpatient cardiac care: Time for a heart to heart?	1
A review of factors affecting patient satisfaction with nurse led triage in emergency departments	1
A qualitative study of patient satisfaction with follow-up cancer care: the case of Hong Kong	2
Assessing patients' satisfaction from the management of pain in the hellenic oncology clinical setting	2
Patient Satisfaction With Anesthesia Care in Taiwan: Development and Validation of a Pilot Version of a Psychometric Questionnaire	5
Influence of mechanism of patient-accessible hospital information system implementation on doctor–patient relationships: A service fairness perspective	3
A comparative study of patients' and nurses' perceptions of the quality of nursing services, satisfaction and intent to revisit the hospital: A questionnaire survey	1
Early Conceptual and Linguistic Development of a Patient and Partner Treatment Satisfaction Scale (TSS) for Erectile Dysfunction	2
Patient satisfaction, characteristics, radiology, and complications associated with attending a specialized government-funded multidisciplinary spinal pain unit	1
Correlation among physical impairments, pain, disability, and patient satisfaction in patients with chronic neck pain	1
A Mental Health Home Visit Service Partnership Intervention on Improving Patients' Satisfaction	1
Patient satisfaction and medication adherence assessment amongst patients at the diabetes medication therapy adherence clinic	1
The Use of Mobile Health Applications to Improve Patient Experience: Cross-Sectional Study in Chinese Public Hospitals"	3
The following papers are Chinese articles:	

门诊服务满意度及诊疗预付费制度认知的研究——杭州市城市居民 300 例调查结果分析	1
实施基本药物制度对门诊患者满意度及就诊意向的影响	7
儿科门诊就诊患儿家属健康教育与满意度调查	3
西北地区某三甲医院住院患者对护理服务的满意度及影响因素分析	3
基层卫生服务患者满意度调查	7
基本医疗保险城乡统筹前后宁夏农村居民的就医选择及满意度分析	2
北京市双向转诊制度实施情况及居民对基层医疗的满意度调查	7
江苏某三甲医院近五年出院患者第三方调查满意度剖析	2
门诊药房药物咨询服务对患者就医满意度的应用分析	2
住院患者满意度调查分析	2
山区农村老年人日常就医选择及满意度	2
医院门急诊患者满意度调查分析	6
急诊患者满意度影响因素调查分析	6
西藏贫困农牧民就医行为及就医满意度调查	2
急诊就诊流程满意度现状分析与改进对策	6
急诊就诊流程满意度调查分析	6
急诊就诊患者满意度调查分析	6
提高门诊导医服务质量对就医患者满意度的影响	3
2014 年度北京市西城区区属公立医院门急诊患者满意度调查与研究	6
北京市医院门诊预约挂号现状及满意度分析	2
卫生人才激励机制改革背景下患者就诊满意度研究	7
门诊多渠道预约挂号平台下患者满意度调查	3
低收入患者住院医疗服务满意度探究——以新疆某济困医院为例	2
一体化流程服务对门诊患者就诊满意度的影响	3
运用 PDCA 强化门诊导医管理 提高患者就诊满意度	3
门诊信息化管理系统运用在患者就诊流程中的价值	3
门诊一站式自助预约挂号服务系统对提高门诊就诊率及满意度的影响	3
创新服务模式在提高门诊患者满意度中的应用	3
门诊自主预约挂号系统对提高患者满意度的影响	3
优化产科门诊服务流程对孕妇就诊时间及满意度的影响	3
“品管圈”活动在提高门诊患者满意度中的作用	3
社区卫生服务中心的就医满意度影响因子分析——基于 W 社区卫生服务站的实证研究	7
社区基层卫生服务满意度的影响因子分析——基于 C 社区卫生服务站的实证研究	7
门诊护理服务满意度的调查与分析	4
社区就诊患者满意度调查及影响因素分析	7
门诊信息化对患者及医务人员工作满意度的影响	3
门诊引导服务对老年患者就诊时间及满意度的影响	3
门诊挂号流程渐进性改进对病人满意度的影响	3

健全门诊导诊制度对提高医院门诊服务水平的影响	3
微信挂号服务模式在儿童医院门诊的应用效果	3
门诊就诊影响因素调查	2
临床检验信息系统建设对门诊病员满意度的影响分析	3
医院专家门诊预约诊疗服务对患者满意度的影响	3
改变门诊就医模式 提高病人满意度	9
影响综合医院门诊护理服务质量因素的研究分析	2
改善门诊医疗服务提升患者满意度	3
利用 1+3 模式改进住院患者就医体验	3
妇幼专科医院住院患者满意度调查研究	2
三甲医院骨科门诊病历患者满意度调查分析	2
日间手术患者就医满意度调查及其影响因素分析	2
门诊患者满意度影响因素研究	9
三城市社区卫生服务机构就诊患者满意度调查	7
门诊病人就医满意度调查	10
门诊就诊患者的变化及满意度调查	10
三级甲等综合医院住院患者就医服务体验及需求的质性研究	2
病人满意度调查的分析与思考	10
兰州市口腔卫生机构就诊患者满意度调查	10
宿迁市医改后居民就医满意度及影响因素调查	2
某大型公立医院患者就医体验的影响因素分析	2
患者就医满意度调查与分析	2
病人满意度及其影响因素研究	2
门诊病人就医影响因素调查分析	10
影响社区卫生服务中心就诊患者满意度的因素分析	7
门诊患者满意度调查对医院管理的几点启示	10
关于某医院门诊就诊满意度调查与分析	10
南宁市社区卫生服务机构就诊居民满意度及其影响因素研究	7
不同挂号方式门诊患者就诊满意度分析	2
门诊预约挂号分时段就诊系统的应用对提高门诊服务效率的作用	2
转诊制度对门诊患者就医满意度的影响分析	2
门诊就医流程患者满意度调查	10
提升住院患者满意度关键环节管理与分析	9
体验经济时代下医院提高患者住院体验的实证分析	2
住院手术患者就医体验调查分析	2
循征护理在提高住院患者满意度中的应用观察	3
Influence of Drug Consultation Service in Outpatient Pharmacy on Patient Satisfaction	3
The influence of the health education on the outpatient satisfaction	3
Associations between waiting times, service times, and patient satisfaction in an endocrinology outpatient department: A time study	6

and questionnaire survey	
The association between patient satisfaction and quality of life in Chinese lung and liver cancer patients	6
A longitudinal analysis on pain treatment satisfaction among Chinese patients with chronic pain: predictors and association with medical adherence, disability, and quality of life	6
Study on Satisfaction Degree with Outpatient Service and Recognition of Prepayment System for Diagnosis and Treatment	10
Level of Satisfaction with Primary Healthcare Services and Influencing Factors in Farmers and Herdsmen in Pastoral Areas of Northern Xinjiang	7
Research of outpatients in Primary traditional Chinese medical hospital	10
Research on Correlations between visit time and patient satisfaction in a variety of investigation ways	10
Factors influencing the outpatient satisfaction and interventions improving the satisfaction	10
Investigation on Satisfaction of Basic Medical Insurance Participants to Different Level Medical Institutions in Hangzhou City	10
Investigation of outpatient satisfaction in tertiary hospital in the city	10
Analysis of satisfaction degree of patients in special requirement outpatient service	6
Investigation of outpatient satisfaction in tertiary hospital	10
Multivariate analysis on satisfaction with health care services of outpatients	7
Investigation of outpatient satisfaction in the County level public hospitals in Ningxia, China	7
Investigation of outpatient satisfaction in the County level public hospitals in Ningxia, China	7
Outpatient satisfaction in county level hospitals in Rong and Luchuan counties, China	7
Investigation and analysis on Outpatients' demand in large general hospitals	10
Analysis of Satisfaction and Determinants Among out-patients of a County Hospital in Hunan	7
Investigation of Outpatient Services Satisfaction in Tertiary Hospital	10
Investigation of the outpatient satisfaction during special outpatient service	6
Investigation on elderly people's satisfaction rate of traditional outpatient process and one stop outpatient service process	10
How to improve the outpatient satisfaction	10
An analysis of outpatients' satisfaction by RSR method	10

Factors influencing outpatient satisfaction and solutions improve the satisfaction	7
Survey and analysis of satisfaction among 1296 outpatients of a dental clinic	6
Study on the experience and influencing factors of outpatients in a Breast specialist outpatient in a tertiary hospital	6
Investigation of the outpatients satisfaction in Chinese traditional medicine hospital in Chongqing	7
Analysis on degree of patients satisfaction and trust to health care in Shanghai	7
Patient satisfaction in two Chinese provinces: rural and urban differences	7
Analysis on degree of patients satisfaction and trust to health care in Shanghai	10
Research on satisfaction of patients and medical personnel based on the new healthcare reform: A case study in Shanghai	7
Analysis on the satisfaction of ecdemic population for medical care in Jiangsu Province	7
Investigation of the relationship between outpatient satisfaction and professionalism and service attitude of doctors	7
Investigation of the outpatient satisfaction in a public hospital in AnShan Hospital	7
Study on influencing factors of satisfaction and intervention measures of outpatients in a maternity and children hospital	6
Investigation of the outpatients satisfaction	6
Outpatient satisfaction and influencing factors in a pediatric hospital	6
The Research on Satisfaction of Different Categories of Customers in Medical Institutions of Shenzhen	7

Supplementary material 4: Titles of include studies

Author and year	Title of the paper
Wenya,Yu et al. 2016.	Determinants and influencing mechanism of outpatient satisfaction: a survey on tertiary hospitals in the People's Republic of China
Liyang,Tang. 2011	The influences of patient's trust in medical service and attitude towards health policy on patient's overall satisfaction with medical service and sub satisfaction in China
Jing Sun et al. 2017<br><br>Laiyang, Wu et al. 2016	Consumer satisfaction with tertiary healthcare in China: findings from the 2015 China National Patient Survey<br><br>Satisfaction of Elderly Patients with Out-patient Services: A Study Based on the 2015 Evaluation of the National Healthcare Improvement Initiative
Jay Pan et al. 2015	Patient dissatisfaction in China: What matters
Jinghua, Li et al. 2016	Patient satisfaction between primary care providers and hospitals: a cross-sectional survey in Jilin province, China
Jinzhu, Xie et al. 2017	Factors associated with outpatient experience of Chinese public hospitals: a cross-sectional study
Chunlei, Han et al. 2012	The outpatient satisfaction investigation in new health reform

	background
Qing Lu et al. 2016	Investigation and Analysis on the Satisfaction Degree of Outpatients in Public Hospitals in Beijing
Jing Zhao et al. 2016	Outpatient satisfaction in tertiary hospital
Wenlong, Hu et al. 2007	Analysis of satisfaction investigation to outpatient service in Xuzhou tertiary hospital
Yanxia, Yang et al. 2015	Analysis on the factors of polyclinic patients' satisfaction
Rong Xu and Xinzhen, Jing. 2004	Study on satisfaction degree in outpatients
Junjie, Sun and Shuangqing, Li. 2018	Experience and Associated Factors of Consultation among Primary Care Outpatients from the Grade A Tertiary General Hospital
Zhanwei, Zhou et al. 2011	Study on satisfaction degree of outpatients in a notched hospital
Caoxin, Bao et al. 2015	Investigation and Analysis of Outpatients' Satisfaction of 2170 Cases
Zhixiang, Teng et al. 2009	Exploration of the associate between the health seeking experience and the outpatient satisfaction
Jing Luan et al. 2013	Relevance of Patient Satisfaction with Health Care to Trust in Residents from Nantong Area
Jianjie Zhang. 2018	Empirical study of the factors influencing the outpatient satisfaction
Chunhui, Ren. 2014	Investigation of the outpatient satisfaction in Changchun
Weiming, Shao et al. 2017	Survey of outpatient satisfaction of a tertiary hospital in Xinjiang province
Ying Zou et al. 2014	Survey of satisfaction of outpatients based on different clinics
Mengzhu, Deng et al. 2013	Investigation and Analysis of Outpatient Satisfaction on Non - technical Quality
Huilan, Luo et al. 2010	Analysis of factors related to outpatients dissatisfaction and strategies improving satisfaction
Zulipiye.et al. 2016	Investigation and Analysis on the satisfaction of outpatients in a general hospital in Xinjiang
Jinping, Shu and Dian Zhou. 2016	Outpatients satisfaction in a tertiary hospital of Anhui province
Kai ling et al. 2009	Survey and strategies of outpatients' satisfaction of senior patients
Yu Li et al 2011	Investigation on Outpatient Service Needs and Satisfaction in Five Tertiary Comprehensive Hospitals in Tianjin City
Qinghua, Zhang and Zhanhe, Liu.	Investigation of the outpatient satisfaction

2011	
Fanzhi, Meng et al.<br>2016	Hospital visit and satisfaction with medical treatment among poor farmers and herdsmen in Tibet
Jun Song et al.<br>2007	Investigation and analysis on the satisfaction of current outpatient service flow
Shi Guo et al.<br>2014	Investigation on the patient satisfaction on the outpatient clinic of a general hospital basis
Xunming, Ji et al<br>2010	Study on patient satisfaction measurement and outpatient service process reengineering based on different treatment chains
